# Deep convolutional neural networks for automated segmentation of brain metastases trained on clinical data

**DOI:** 10.1186/s13014-020-01514-6

**Published:** 2020-04-20

**Authors:** Khaled Bousabarah, Maximilian Ruge, Julia-Sarita Brand, Mauritius Hoevels, Daniel Rueß, Jan Borggrefe, Nils Große Hokamp, Veerle Visser-Vandewalle, David Maintz, Harald Treuer, Martin Kocher

**Affiliations:** 1 University of Cologne, Faculty of Medicine and University Hospital Cologne, Department of Stereotactic and Functional Neurosurgery, Cologne, Germany; 2 University of Cologne, Faculty of Medicine and University Hospital Cologne, Department of Diagnostic and Interventional Radiology, Cologne, Germany

**Keywords:** Brain metastasis, Magnetic resonance imaging, Deep learning, Segmentation, Stereotactic radiosurgery

## Abstract

**Introduction:**

Deep learning-based algorithms have demonstrated enormous performance in segmentation of medical images. We collected a dataset of multiparametric MRI and contour data acquired for use in radiosurgery, to evaluate the performance of deep convolutional neural networks (DCNN) in automatic segmentation of brain metastases (BM).

**Methods:**

A conventional U-Net (cU-Net), a modified U-Net (moU-Net) and a U-Net trained only on BM smaller than 0.4 ml (sU-Net) were implemented. Performance was assessed on a separate test set employing sensitivity, specificity, average false positive rate (AFPR), the dice similarity coefficient (DSC), Bland-Altman analysis and the concordance correlation coefficient (CCC).

**Results:**

A dataset of 509 patients (1223 BM) was split into a training set (469 pts) and a test set (40 pts). A combination of all trained networks was the most sensitive (0.82) while maintaining a specificity 0.83. The same model achieved a sensitivity of 0.97 and a specificity of 0.94 when considering only lesions larger than 0.06 ml (75% of all lesions). Type of primary cancer had no significant influence on the mean DSC per lesion (*p* = 0.60). Agreement between manually and automatically assessed tumor volumes as quantified by a CCC of 0.87 (95% CI, 0.77–0.93), was excellent.

**Conclusion:**

Using a dataset which properly captured the variation in imaging appearance observed in clinical practice, we were able to conclude that DCNNs reach clinically relevant performance for most lesions. Clinical applicability is currently limited by the size of the target lesion. Further studies should address if small targets are accurately represented in the test data.

## Keypoints

- Deep learning allows accurate detection and segmentation of brain metastases
- Performance of the algorithm depends on the size of the target lesion
- Combination of differently trained networks improves overall performance

## Introduction

Stereotactic radiosurgery (SRS) is considered standard of care in patients with one or multiple brain metastases (BM) of limited size and number [1]. The high incidence of BM [2] and the necessity to manually contour each lesion for definition of the planning target volumes of radiosurgery results in high workload for the attending physicians. However, contouring of planning CT and MR images generates a large amount of clinically representative imaging and contour data. This data can be used to train deep convolutional neural networks (DCNNs) which have been applied to various tasks in medical imaging including segmentation in the recent years [3]. If trained properly, DCNNs could not only increase clinical efficacy by speeding up detection and contouring of BM but could also increase inter-rater agreement [4].

The first application which produced state-of-the-art results in automated segmentation of BM in MRI was published in 2015 by Losch et al. [5]. Since then, a large variety of network architectures for deep learning including GoogLeNet [6], CropNet [7], DeepMedic [8] and En-DeepMedic [9] have been tested. A common limitation is the high number of false positives and the small sample sizes used for training. Also, the performance of most of the algorithms heavily depended on the volume of the target lesion. Dikici et al. identified this problem and developed an algorithm which was trained and performed well only on small BM (mean volume of 160 mm^3^) [7], which suggests that a single model is unlikely to solve the segmentation problem for metastases of arbitrary size.

One of the most commonly used network architectures is the so-called U-Net [10]. Recently, Isensee et al. demonstrated how this relatively simple architecture combined with a robust training scheme achieved state of the art performance on different challenges in segmentation of medical images [11]. A slightly modified version of the architecture was used by Kickingereder et al. to not only segment brain lesions, but also to precisely track treatment response by assessing reductions in diameters of the corresponding automated segmentation [12]. The capability of this approach is underlined by the fact that the dataset used in their work not only included glioblastomas but also lower-grade gliomas which typically differ markedly in appearance from their malignant counterparts, which suggests that it may also be useful for segmentation of brain metastases that differ in morphology from both types of glioma.

In the present work we applied a state-of-the-art U-Net to a large and clinically representative dataset of BM collected from a group of patients, who had been treated by SRS during a period of 6 years. This allowed us to accurately assess the applicability of such algorithms for automated segmentation in radiation therapy planning.

## Methods

### Data acquisition

This retrospective single-center study was approved by the institutional review board and informed consent was waived. Brain images from a cohort of patients with cerebral metastases treated by SRS between 2013 and 2019 were used to train a DCNN. All patients underwent SRS by means of the CyberKnife® (Rel. 9.5, Accuray) stereotactic treatment system. For treatment planning MR images including contrast-enhanced T1-weighted (T1c), T2-weighted and T2-weighted, fluid-attenuated (FLAIR) images were registered to the planning CT. Patients were excluded from this study if any of those sequences was not acquired for treatment planning. Target structures (planning target volumes, PTV) were manually delineated on the MR images by board-certified neurosurgeons or radiation oncologists. Usually, the contours were first outlined on the axial T1c images at the rim of the contrast enhancement. In cases with atypical or no enhancement, contouring was performed using the information from the other T1c slice orientations and image types. The original images and contour data were restored from the archive and randomly split into a set for training and testing DCNNs. To some extent, the training data set was only weakly labelled since not all lesions visible on the MR at the time of treatment qualified for SRS and were thus not contoured. To properly evaluate the performance of the algorithm, the test set was revised by a senior radiation oncologist (M.K., 25 yrs. experience) who delineated every present lesion. The MR-images were preprocessed by applying a deep-learning-based tool for brain extraction HD-BET [13] and by resampling the image and contour data to isotropic voxels with a size of 0.89mm^3^. Finally, z-score intensity normalization was applied to all images.

### Architecture of the deep convolutional neural network

For the DCNN’s architecture, a conventional U-Net (cU-Net) [10] and a modified U-Net (moU-Net) with multiple outputs were implemented using Python 3.6.5 (Python Software Foundation, Beaverton, Oregon, USA) and TensorFlow 1.11 [14]. The U-Net is characterized by an encoder which extracts low-level representations of the input data and which is connected to a decoder which reconstructs the corresponding label map with skip connections between intermediary stages of both modules. Since its original inception, several optimizations in regards to the network structure and the training process were applied to the U-Net [11]. Modifications to the network included adaptation of residual connections [15], instance normalization [16] and the usage of leaky rectified linear units (ReLU) as activation functions. Robust training was achieved through sampling of volumetric patches and image augmentation.

**Fig. 1:**
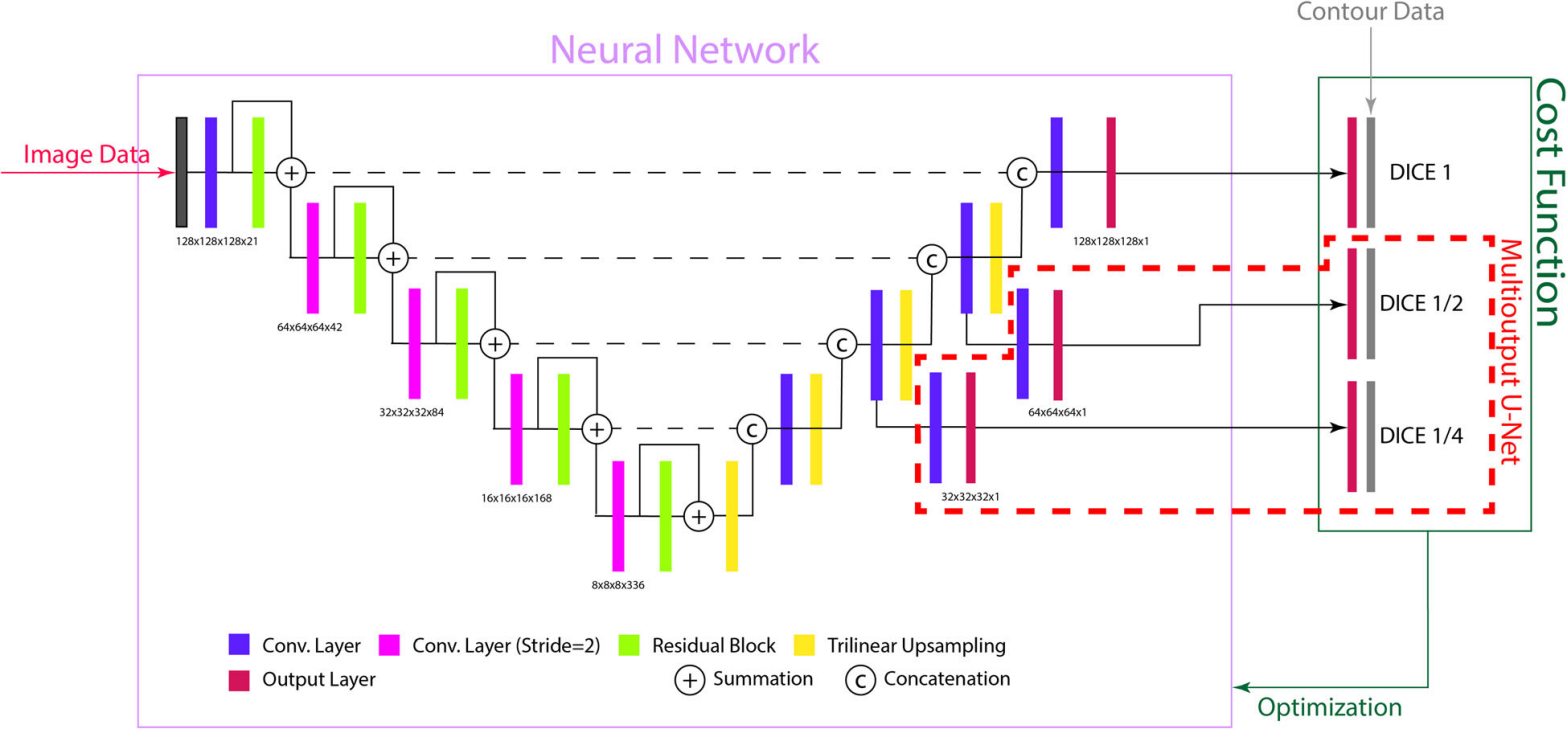
Architecture of the trained U-Net. All convolutions used filters with a kernel size of 3. Before all convolutions, instance normalization and the activation function (leaky ReLU) were applied to the input. The residual block contained two such convolutions. Downsampling in the encoding layer was realized using a convolution with a stride of 2. In the output layers the sigmoidal function is applied to the DCNN’s output. For the moU-Net, two intermediate output layers are added (dashed red lines). The original contour data is then used to compute the cost function

The moU-Net was proposed by Kickingereder et al. and demonstrated high performance for segmentation of glioblastoma and lower-grade glioma [12]. While the network’s encoder and decoder layers are identical to the conventional U-Net, the moU-Net adds additional output layers to the decoder. The motivation of this procedure is to ensure that the network uses its entire receptive field. During training, the additional outputs are used as auxiliary loss layers by comparing them to downscaled versions of the reference label data. Both the cU-Net and the moU-Net are depicted in Fig. 1.

### Training

The training process was adopted from the original paper describing the moU-Net, see [12] for a detailed description. During training, the networks were presented with randomly sampled volumetric patches with a size of 128^3^ pixels from all three imaging sequences (T1c, T2 and FLAIR) resulting in a volumetric patch with three channels. To improve the network’s generalizability, image augmentation through random rescaling [factor 0.75 or 1.25], random rotation [− 45 °, + 45°], random flipping [50% along all axes], elastic deformation [17] and channel-wise gamma augmentation [0.8, 1.5] was employed. The networks were optimized using the soft dice loss formulation:

$$ l\left(P,T\right)=1-2\frac{\sum \limits_i{p}_i{t}_i}{\sum \limits_i{p}_i+\sum \limits_i{t}_i} $$

Here, *p*_*i*_ is an element contained in the network’s output layer *P* and *t*_*i*_ is the corresponding label in the ground-truth segmentation *T*. For the moU-Net, the output of the auxiliary layers was compared to down-sampled (factor 1/2 and 1/4) versions of the ground truth and a weighted sum of the losses were calculated:

$$ L=0.25\ {l}_{\frac{1}{4}}+0.5\ {l}_{\frac{1}{2}}+1\ {l}_1 $$

Both types of networks were trained for 450 epochs with 200 iterations with an exponentially decaying learning rate: *a*_*epoch*_ = 10^−4^ ∗ 0.99^*epoch*^ using a batch size of 2. The networks’ weights were optimized using Adam [18] and L2-regularization (*β* = 0.0001). All models were trained employing four-fold cross-validation resulting in an ensemble of four models used for inference on the test set [19].

### Additional training on small metastases

Since the performance of the segmenting DCNNs depends on the target lesions size [6; 7], additional training on a subsample of the data containing lesions smaller than 0.4 ml was performed. The resulting U-Nets trained on data with small lesions (sU-Net) were generated by further training of the pre-trained conventional U-Nets. In cases containing lesions above and below the threshold of 0.4 ml, labels contained in the lesions above the threshold were not considered for loss calculation.

### Inference

The performance of the three network types was evaluated by comparing the segmentations generated by the DCNN based on image data from the test set to the corresponding ground truth represented by the manual segmentations. The predictions were gained based on whole image series. The images were presented to each of the four trained networks contained in the respective trained ensemble. Test time data augmentation was employed by flipping the images along all three axes and averaging the output of the final layer [20]. Finally, the segmentations generated by each network of the ensemble were merged by summation or majority voting. For post-processing, any remaining structure smaller than the smallest BM (0.006 ml) contained in the dataset was removed from the generated segmentation. This procedure was performed for the cU-Net, moU-Net and the sU-Net. The performance of the combined model was assessed in two configurations employing different ensembling techniques for the underlying networks. For NetMV the cU-Net, sU-net and moU-Net each used majority voting as ensembling technique before combining the three resulting automatic segmentations, while NetSUM used summation as ensembling technique.

### Statistical analysis

To assess the quality of the resulting segmentations, multiple metrics were employed. The dice similarity coefficient (DSC) measures the overlap with the ground truth (ranging from 0 for no overlap to 1 for perfect overlap) per patient. The algorithm’s performance in detecting individual metastases was measured by sensitivity (detected metastases divided by all metastases contained in ground truth), specificity and average false positive rate (AFPR). The F1-score combines sensitivity and specificity into a single metric by calculation of their harmonic mean and was used to find the most balanced model. A metastasis was considered detected when the DCNN’s segmentation overlapped with the corresponding ground truth segmentation. The degree of overlap was measured as mean DSC per metastasis. Differences of the tumor volumes between the prediction and the ground truth were additionally measured using Bland-Altman plots [21] and the concordance correlation coefficient (CCC) [22]:

$$ CCC=\frac{2\rho {\sigma}_T{\sigma}_P}{\sigma_T^2+{\sigma}_P^2+{\left({\mu}_T-{\mu}_P\right)}^2} $$

where *μ*_*T*_ and *μ*_*P*_ are the means for predicted and the ground truth tumor volumes, *σ*_*T*_ and *σ*_*P*_ the respective variances and *ρ* the correlation coefficient between both. All statistical methods except for the CCC, which was calculated using R (version 3.3.2; R Development Core Team) with the “epiR” package, were implemented in Python 3.6. Differences in predictive performance between types of target lesions was tested using one-way ANOVA [23].

## Results

### Patient characteristics

From a list of 1400 SRS treatments between April 2012 and June 2019, 835 treatments of brain metastases were identified. If a patient was treated multiple times, only the first treatment was considered for this study, reducing the dataset to 634 patients. 561 of those datasets could successfully be restored from the imaging archive. Finally, a dataset of 509 patients (1223 brain metastases) with the required T1c, T2 and FLAIR images and contour data was used for this study (Table 1). The cohort was randomly split into a set of 469 and 40 patients for training and testing the algorithm. A typical distribution [24] of primary tumor types was observed with lung cancer being the most frequent primary tumor (54%/50%), followed by melanoma (18%/23%) and breast cancer (10%/10%). All imaging was performed on MR scanners manufactured by Philips Medical Systems and almost all series (99.2%) were acquired using a magnetic field strength of 3 T. Median size of the BM was 0.31 ml (training) and 0.41 ml (test) with large variability in size demonstrated through interquartile ranges (IQR) of 1.3 ml and 1.6 ml respectively. In total, 524 lesions from 257 patients were smaller than 0.4 ml and were used to train the sU-Net.

**Table 1:** Characteristics of the patient cohort enrolled in this study

	Train	Test
No. of Patients	469	40
Gender (Female/ Male)	244 / 225	26 /14
Mean Age	61	62
No. of Metastases	1149	83
Metastases < 0.4 ml (No. of Patients)	524 (257)	47 (24)
Median Lesion Size	0.31 ml	0.47 ml
IQR	(0.09–1.32 ml)	(0.14–1.82 ml)
Mean Lesion Size	1.29 ml	1.92 ml
**Primary Tumor**		
Lung	255	20
Melanoma	85	9
Breast	48	4
Other	66	6
Mixed	9	0
CUP	6	1
**Year of Treatment**		
2013	13	1
2014	78	2
2015	98	6
2016	97	14
2017	97	8
2018	63	6
2019	23	3
**MR Device (Field Strength)**		
Ingenia (3.0 T)	372	37
Ingenia (1.5 T)	2	0
Archieva (3.0 T)	93	3
Intera (1.5 T)	2	0

### Network performance

**Fig. 2:**
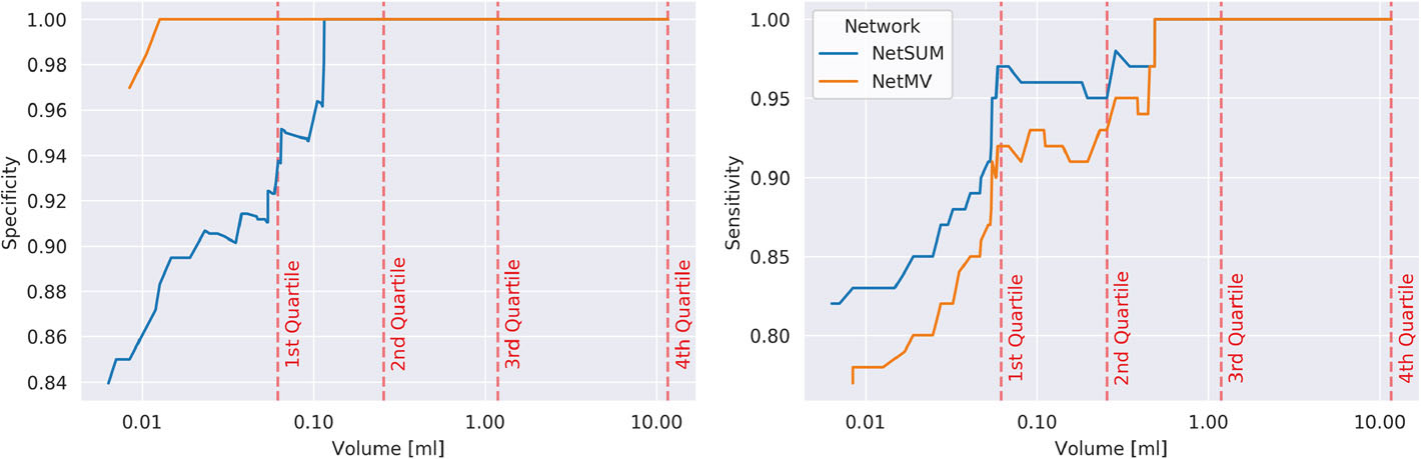
Sensitivity and Specificity of the developed networks plotted against the minimum volume of the considered target lesions. Dashed lines depict the four quartiles (*Q*_*i*_) of the measured volumes of target lesions in the test data (*Q*_1_ = 0.06 ml, *Q*_2_ = 0.29 ml, *Q*_3_ = 1.29 ml, *Q*_4_ = 8.05 ml). The largest drop in both sensitivity and specificity is observed for lesions smaller than 0.06 ml. At this threshold the sensitivities and specificities are 0.97/0.92 and 0.92/1.00 for NetSUM and NetMV respectively

The combination of all three networks types with ensemble building through summation (NetSUM) achieved the highest sensitivity of 0.82, while the combination based on majority voting (NetMV) demonstrated a balanced performance of high sensitivity (0.77) while also retaining high specificity (0.96) measured by the overall highest F1-score of 0.85 (Table 2). Since performance of the algorithm depends on the used test set and to allow fair comparison to previous work, we furthermore report the results for different volumes of target lesions (Fig. 2). A large drop in performance is observed for lesions smaller than 0.06 ml (25% of test data). Sensitivities and specificities for BMs above this threshold are 0.92 and 1.00 for NetMV and 0.97 and 0.94 for NetSUM respectively.

**Table 2:** Performance of the algorithms by network type and type of ensemble building. SUM: summation, MV: majority voting

DCNN Type	Ensemble	Sensitivity	Precision	F1-Score	Sensitivity Small BM	AFPR	Mean DSC	DSC
	Method							per Lesion
moU-Net	SUM	0.71	0.89	0.79	0.51	0.18	0.71	0.74
cU-Net	SUM	0.71	0.94	0.81	0.51	0.1	0.7	0.73
sU-Net	SUM	0.53	0.85	0.65	0.68	0.2	0.27	0.61
**NetSUM**		**0.82**	0.83	0.82	**0.7**	0.35	0.7	**0.74**
moU-Net	MV	0.65	0.96	0.78	0.43	0.05	0.71	0.73
cU-Net	MV	0.63	1	0.77	0.4	0	0.69	0.73
sU-Net	MV	0.43	0.95	0.59	0.62	0.05	0.21	0.52
**NetMV**		0.77	**0.96**	**0.85**	0.64	**0.08**	**0.71**	0.71

*DSC* dice similarity coefficient, *AFPR* average false positive rate, *F1-score* combines sensitivity and specificity into a single metric by calculation of their harmonic mean in order to find the most balanced model

**Fig. 3:**
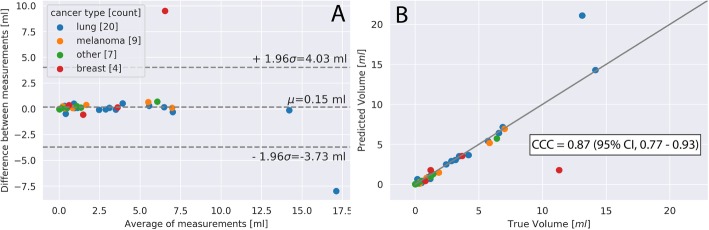
**a:** Bland-Altmann plot visualizing agreement between manually delineated ground truth and automatic segmentation by DCNN per patient. The middle horizontal line is drawn at the mean difference (0.15 ml) between both measurements and the lines below and above at the limits of agreement (95% CI). **b:** Volume predicted by DCNN plotted against manual segmentation. The concordance correlation coefficient (CCC) measuring deviation from the diagonal line depicting perfect agreement between both volumes was 0.87

**Fig. 4:**
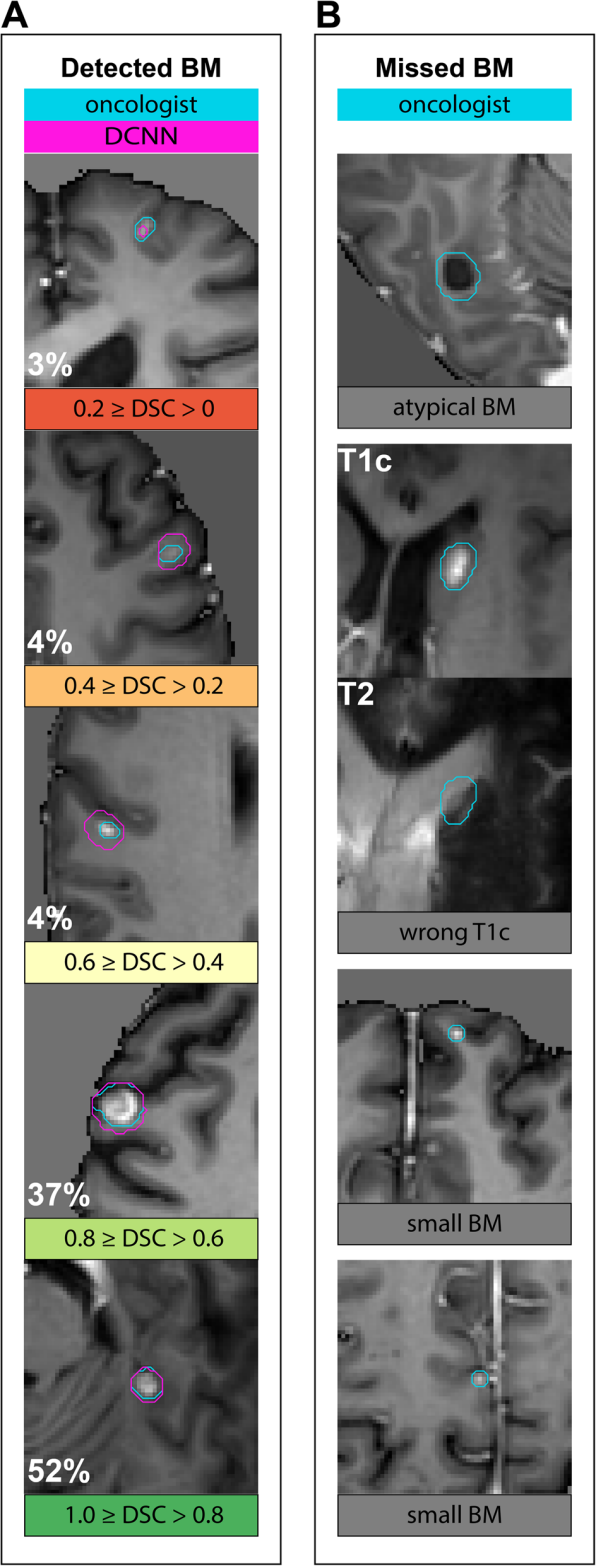
Samples of T1c images (if not otherwise specified) containing ground truth segmentations (blue lines) and segmentations by DCNN (purple lines). **a:** Randomly selected samples of detected lesions. Number in bottom left of each image is the percentage of segmentations with similar quality of segmentation measured by DSC. **b:** Samples of undetected lesions. *Atypical BM:* Largest undetected lesion with minor contrast-uptake in rim. *Wrong T1c:* Second-largest undetected lesion where T1c images came from a different study than T2 and FLAIR images. *Small BM:* Randomly selected samples of undetected lesions

Agreement between the total tumor volumes predicted using NetSUM and the ground truth volumes, visualized in a Bland-Altman plot (Fig. 3 a) and measured by a CCC of 0.87 (95% CI, 0.77–0.93), was excellent (Fig. 3b). Mean difference between both volumes was 0.15 ml (95% CI, − 3.73 ml – 4.03 ml). Samples containing detected and missed lesions are depicted in Fig.4. Quality of segmentations was above a 0.8 DSC for 52%, 0.6–0.8 DSC for 37% and below a 0.6 DSC for 11% of all detected lesions. Undetected lesions had volumes between 0.017 ml and 0.06 ml, with two exceptions (Fig. 4b): one lesion with uncharacteristic contrast-uptake (0.489 ml) and one where image sequences were acquired during different studies (0.288 ml). We also tested if the mean DSC varies between the types of primary tumors (Lung, Melanoma, Breast and other) using one-way ANOVA and no significant influence (*p* = 0.60).

### Ablation study

**Fig. 5:**
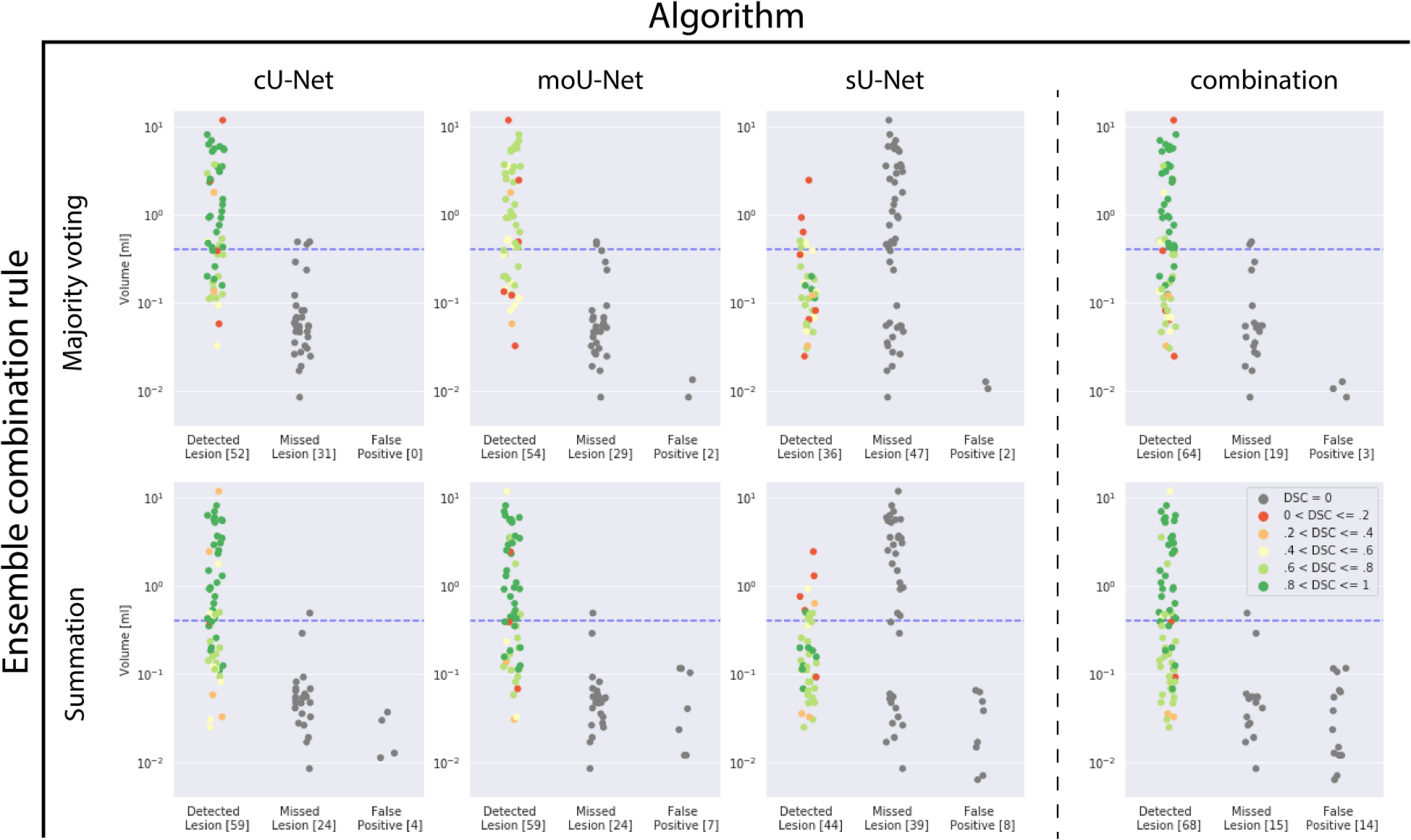
Results per lesion for all algorithms (cU-Net, moU-Net, sU-Net and their combination) and ensemble building through summation and majority voting. A lesion in the test set (40 patients, 83 lesions) was considered detected if it overlapped with a segmentation produced by the respective algorithm. The degree of overlap and thus the quality of the segmentation was assessed using the dice similarity coefficient (DSC). The dashed blue line is the threshold at which a lesion was defined as small (< 0.4 ml) and thus used to train the sU-Net

To assess how the cU-Net, moU-Net, sU-Net perform individually the networks were each evaluated on the test set (Fig. 5, Table 2). For all networks, using summation as ensembling technique resulted in a higher sensitivity, but lower specificity compared to majority voting. The highest overall sensitivity of 0.71 was achieved by both the cU-Net and the moU-net, where the cU-Net had a higher specificity (0.94) compared to the moU-net (0.89) and a higher F1-score of 0.81. Majority voting reduced sensitivity to 0.63 and 0.65, but increased specificity to 1.00 and 0.96 for the cU-Net and moU-Net respectively. While the sU-Net had the lowest overall sensitivities (0.53 summation/0.43 majority voting), it outperformed the other networks in sensitivity for smaller lesions (0.68 / 0.52).

## Discussion

In this work, a deep learning-based algorithm was successfully trained to automatically segment brain metastases. The final model was tested in two configurations, with the more sensitive one reaching an overall sensitivity of 0.82. Compared to previous studies the same model was highly specific with a specificity of 0.83 [7]. The data contained a broad range of metastases in regards to both primary cancer type and volume. While primary cancer type had no influence on the DCNNs performance, we observed a reduction for lesions smaller than 0.06 ml. Above this threshold the sensitivity and specificity of the algorithm were 0.97 and 0.94 respectively. This underlines how important usage of clinical representative data is to properly evaluate such algorithms. Having this current lower limit in mind enables controlled clinical usage for automated segmentation in radiation therapy. Quality of the automatically segmentations was very high (1 ≥*DSC*≥ .8) for 52% of lesions and high (.8 ≥*DSC*≥ 6) for 37% of lesions, which demonstrated that this algorithm can provide a robust segmentation for therapy planning. In addition to high performance in both detection and segmentation, usage of GPUs (NVIDIA RTX 2080ti) allows for rapid computing, resulting in a computation time between 4 and 5 min for loading, preprocessing and segmenting the image data.

Regarding optimization of the DCNN, our results in the ablation study show the commonly used cU-Net performing similarly to the moU-Net, demonstrating that the conventional U-Net is already well optimized. For small BMs, the sensitivity of both the cU-Net and the moU-Net dropped to values ranging from only 0.40 to 0.51. Addition of the sU-Net, which was a conventional U-Net trained on a subsample containing only small BMs with a sensitivity for small lesions of 0.62–0.68, to the ensemble improved the sensitivity of the combined model considerably. Interestingly, the sU-Net didn’t detect larger BMs (see Fig. 2) which indicates that a combination of networks trained on different subsamples of the data is needed for the overall model to fully generalize. The higher false-positive rate of the sU-Net compared to the other models, suggests that subsamples need to be of sufficient size to ensure high specificity.

Comparability of models to other work is often difficult due to differences in how the data was generated and rated. Overall, our model was slightly less sensitive in detecting metastases compared to other models (Table 3). The model with the highest sensitivity of 0.93 by Charron et al. [8] also used data generated for SRS, but their dataset included more larger lesions, on which a DCNN typically performs well. Dikici et al. [7] used a set of homogeneously small lesions and achieved results similar to ours. This confirms our findings that a specific dataset could allow DCNNs to perform similar on small lesions and probably on atypical lesions (see Fig. 4 b) compared to average-sized and typically configured BM. With regard to specificity, our model outperforms all other models by a magnitude, which demonstrates that recent optimizations of the structure and training scheme of the U-Net could eventually allow to use it in a clinical context.

**Table 3:** Comparison of deep learning based segmentation studies for brain metastases (adapted from Dikici et al. [7]). AFPR: average false positive rate

Study	Patients	Multiparametric MRI	Dedicated Test Set	Mean BM volume (mm)	Median BM volume (mm)	Sensitivity	AFPR
Losch et al. [5]	490	no	yes	NA	NA	0.83	7.7
Charron et al. [8]	182	yes	yes	2400	500	0.93	7.8
Grøvik et al. [6]	156	yes	no	NA	NA	0.83	8.3
Dikici et al. [7]	158	no	no	159.6	50.4	0.9	9.12
Present paper	509	yes	yes	1290 (train)	310 (train)	0.77–0.82	0.08–0.35
				1920 (test)	470 (test)		

A very recent study by Xue et al. [25] is not listed in the comparison in Table 3. They also used data gained from SRS and claimed an accuracy of 100% on their data. The median volume of lesions in their dataset was 2.22 ml which is more than quadruple the median size in our training (0.31 ml) and test (0.47 ml) set and also larger than any other dataset in our comparison. The smallest lesion in their training set had a size of 0.07 ml, while 26% of lesions in our test set were in fact smaller. Additionally Xue et al. [25] calculated sensitivity and specificity of their model per pixel and nor per lesion, which heavily favors larger lesions. The used dataset also has a typical distribution of primary cancer types, which we used to show the robust performance of the DCNN for different primary tumors. Overall, we are confident that our study is a realistic and thus clinically relevant observation of how a DCNN is expected to perform.

Our results suggest that mining image and contour data acquired for SRS is a viable option for in-house development of powerful deep learning algorithms. As demonstrated on other non-clinical datasets before [26], the learning process is robust to partially missing labels. A benefit of in-house development is the option to modify the algorithm, e.g. by changing the method used to ensemble the trained networks from majority voting (high specificity) to summation (high sensitivity) depending on the task. Trained networks can furthermore easily be shared, which would boost reproducibility [27] and overall performance by generating an ensemble of models trained at various sites.

By including an established deep learning-based tool for brain extraction [13] in our preprocessing pipeline it was also demonstrated that artificial intelligence is reaching a point where algorithms can be used reliably in well-defined tasks. Such pipelines have to be implemented and supervised carefully to ensure that errors don’t propagate through the chain of algorithms. The present pipeline can eventually be expanded to include automatic classification of a BMs histology [28], prediction of treatment response [29] or to directly influence the treatment e.g. through dose optimization [30].

A limitation of this study is that only patients eligible for SRS were considered. While SRS is a viable option for patients with multiple small to medium-sized metastases, exemplified by a treatment of nine lesions contained in the present cohort, our dataset and thus the developed network could be biased due to a fraction of patients undergoing other treatments such as whole brain radiation therapy.

## Conclusion

In summary, we demonstrated that neural networks developed using data acquired during clinical practice are capable of automated image segmentation. We also pointed out that the performance of such algorithms still depends on the size of the target lesion. Further studies should ensure that performance on such lesions is properly measured, to ensure clinical applicability. For the large majority of target lesions however, automated segmentation for treatment planning is already a viable option to provide a robust baseline segmentation.

## Data Availability

Patient data currently cannot be made available. Implemented methods can be inspected onsite.

## Abbreviations

*BM*: Brain metastasis
*DCNN*: Deep convolutional neural network
AFPR: Average false positive rate
DSC: Dice similarity coefficient
CCC: Concordance correlation coefficient
SRS: Stereotactic radiosurgery
T1c: T1-weighted contrast-enhanced MRI
FLAIR: Fluid-attenuated inversion recovery
